# 5‐Chloro‐2,4‐dihydroxypyridine, CDHP, prevents lung metastasis of basal‐like breast cancer cells by reducing nascent adhesion formation

**DOI:** 10.1002/cam4.1265

**Published:** 2018-01-22

**Authors:** Junji Itou, Hiroshi Tsukihara, Mamoru Nukatsuka, Masakazu Toi, Teiji Takechi

**Affiliations:** ^1^ Department of Breast Surgery Graduate School of Medicine Kyoto University 54 Shogoin‐Kawahara‐cho, Sakyo‐ku Kyoto 606‐8507 Japan; ^2^ Translational Research Laboratory Taiho Pharmaceutical Co., Ltd. 224‐2 Ebisuno Hiraishi, Kawauchi‐cho Tokushima 771‐0194 Japan

**Keywords:** 5‐chloro‐2,4‐dihydroxypyridine, basal‐like breast cancer, gimeracil, metastasis, S‐1

## Abstract

A drug for metastasis prevention is necessary. The orally administered anticancer drug S‐1 contributes to cancer therapy. In a mouse xenograft model of metastatic breast cancer from our previous study, the administration of S‐1 inhibited lung metastasis. However, the mechanism of inhibition remains elusive. S‐1 contains 5‐chloro‐2,4‐dihydroxypyridine (CDHP), which does not have the antigrowth activity, but prevents the degradation of 5‐fluorouracil, an anticancer reagent. In this study, we found that CDHP treatment shrinks cell morphology in metastatic basal‐like breast cancer cell lines. Wound healing assays showed reduced cell migration in CDHP‐treated cells. At the molecular level, CDHP treatment reduced the number of nascent adhesions, whereas the number of mature focal adhesions was not changed. These findings indicate that CDHP impairs focal adhesion formation, which results in a reduction in cell migration. For the *in vivo* metastasis assay, we used a highly lung‐metastatic cell line. We xenografted them into immunodeficient mice, and administered CDHP. To determine whether CDHP prevents metastasis, we measured the weights of harvested lungs. The results showed that the lung weights of the CDHP‐treated animals were not significantly different compared to the no‐tumor controls, whereas the vehicle group showed a number of metastatic foci and an increase in lung weight. These observations indicate that CDHP administration prevents metastasis. This study reveals a novel effect of CDHP for lung metastasis prevention. Our findings may facilitate the establishment of future metastasis prevention therapies.

## Introduction

Cancer cells migrate from the primary site to other parts of the body and generate metastatic foci, which cause death. Therefore, therapy for metastasis prevention is required. Given that the enhancement of cell migration is necessary for metastasis, inhibition of cancer cell migration may prevent metastasis.

Basal‐like breast cancer cell lines have a high migratory ability, and are a useful model for studying metastasis. They show an elongated morphology in cell culture. Focal adhesion is required for cell migration. In cells having an elongated morphology, focal adhesion is observed at the front and the rear regions of a cell 1. At the first step of focal adhesion formation, membrane‐bound adhesion molecules, called integrins, form a heterodimer of alpha and beta subunits 1. Subsequently, focal adhesion molecules, such as focal adhesion kinase and paxillin, are recruited to the cytoplasmic tail of integrin molecules 2. This complex is called a nascent adhesion or a focal complex 2, 3. For focal adhesion formation, nascent adhesions appear at the leading edge of a cell, and subsequently, some of them become mature focal adhesions 3. Vinculin localizes to an adhesion complex, and the actin cytoskeleton is organized, bringing about focal adhesion maturation 2, 3. Mature focal adhesions disassemble, and new focal adhesions assemble 1, 4. If focal adhesion formation is impaired, cell migration is reduced.

The orally administered fluoropyrimidine, S‐1, is composed of a 1:0.4:1 molecular ratio of tegafur, a masked form of 5‐fluorourcil: 5‐chloro‐2,4‐dihydroxypyridine, a potent dihydropyrimidine dehydrogenase (DPD) inhibitor (CDHP): potassium oxonate, an inhibitor of 5‐fluorourcil phosphorylation in the gastrointestinal tract 5, 6, 7. Both CDHP and potassium oxonate have no antigrowth activity, whereas 5‐fluorourcil inhibits growth. CDHP inhibits 5‐fluorourcil degradation in liver, which results in the maintenance of the 5‐fluorourcil concentration in the body. S‐1 is used for therapies for various cancers, such as breast, gastric, and pancreatic cancers 8, 9, 10, 11, 12. The previous study has reported that S‐1 administration inhibits metastasis of a high lung‐metastatic subline of a basal‐like breast cancer cell line, MDA‐MB‐231, in a mouse xenograft model 13. Although 5‐fluorourcil inhibits cancer growth, how S‐1 prevents metastasis remains elusive. In this study, we aimed to reveal the mechanism of antimetastatic activity of S‐1. We focused on CDHP, because tegafur has antigrowth activity and it is difficult to analyze only antimetastatic activity, and potassium oxonate mainly localizes in gut after oral administration of S‐1 and does not seem to affect the activities of cancer cells generated in other organs. Here we show a novel effect of CDHP on metastasis prevention. Our findings may contribute to the development of future metastasis inhibitors.

## Materials and Methods

### Cell culture

Basal‐like breast cancer cell lines, SUM159 and MDA‐MB‐231 were obtained from Asterand (Detroit, MI) and the American Type Culture Collection (Manassas, VA), respectively. MDA‐MB‐231 LLM cells were established from metastasized MDA‐MB‐231 cells 13. Short tandem repeat analyses were performed for cell authentication in May 2017, and the results showed no contamination and no alteration of SUM159 or MDA‐MB‐231 cells. MDA‐MB‐231 LLM cells were considered to be MDA‐MB‐231 cells. SUM159 cells were maintained with Ham's F‐12 nutrient mixture containing 5% FBS, 5 *μ*g/mL insulin, 1 *μ*g/mL hydrocortisone, and 10 mmol/L HEPES. MDA‐MB‐231 cells were maintained with RPMI‐1640 containing 10% FBS. CDHP (Tokyo Chemical Industry, C2243, Tokyo, Japan) was dissolved in dimethyl sulfoxide (DMSO). Cells were treated with CDHP for 3 days, and analyzed. For the wound healing assay, a confluent culture was manually scratched with a 200 *μ*L tip. For the invasion assay, cells were treated with CDHP for 2 days. Twenty thousand cells were plated into matrigel‐coated boyden chamber, and incubated for 24 h. In the wound healing and the invasion assays, to inhibit cell proliferation during incubation, 1 *μ*g/mL of mitomycin C was added. To analyze cell migration and invasion, cells were stained with crystal violet.

### Immunocytochemistry

For immunocytochemistry, cells were plated on a glass‐bottom chamber slide (Matsunami Glass, SCS‐008, Osaka, Japan). After fixation, cells were washed, and permeabilized with 0.5% Triton X‐100. Subsequently, cells were washed, and incubated with blocking buffer that contained 5% goat serum and 1% BSA. After blocking, cells were incubated with the primary antibodies, mouse anti‐vimentin antibody (Sigma, V6389, St. Louis, MO, 1:20 dilution), mouse anti‐E‐cadherin antibody (BD Biosciences, 610182, San Jose, CA, 1:50 dilution), rabbit anti‐phospho‐paxillin antibody (Cell Signaling Technology, 2541, Danvers, MA, 1:20 dilution) and mouse anti‐vinculin antibody (Sigma, V9131, 1:200 dilution). Cells were washed, and stained with the secondary antibodies, rabbit anti‐mouse IgG antibody conjugated to Alexa 568 (Life Technologies, A11061, Carlsbad, CA, 1:200 dilution), goat anti‐rabbit IgG antibody conjugated to Alexa Fluor 546 (Life Technologies, A11010, 1:1000 dilution) and goat anti‐mouse IgG conjugated to Alexa Fluor 488 (Life Technologies, A11001, 1:1000 dilution). In cells immunostained for vimentin and E‐cadherin, nucleus was counterstained with DAPI. Fluorescent images were obtained using an all‐in‐one microscope (Keyence, BZ‐9000, Osaka, Japan).

### *In vivo* metastasis assay

All animal study procedures were performed according to the protocols and guidelines of the Institutional Animal Care and Use Committee of Taiho Pharmaceutical Co., Ltd. Ethical approval was obtained prior to the execution of animal experimentation. Five‐week‐old female C.B‐17/Icr *scid*/*scid* mice were purchased from CLEA Japan (Tokyo, Japan). All mice were housed under specific pathogen‐free conditions. A piece of tumor tissue of the MDA‐MB‐231 LLM cells was xenografted into the mammary fat pad. The components of S‐1 were synthesized at Taiho Pharmaceutical Co., Ltd. Hydroxypropyl methylcellulose (vehicle), CDHP or S‐1 was administered orally every day. Paclitaxel (Tokyo Chemical Industry, P1632) was dissolved in 8.3% Cremophor EL and 8.3% ethanol in saline, and administered intravenously. Tumors at the xenografted sites were excised on day 24 under analgesic treatment with pentobarbital and meloxicam, and on day 64 mice were killed and whole lungs were harvested.

### Statistical analyses

For the analyses of the adhesion signals, Student's *t*‐test was used. For the wound healing assay, the invasion assay and the *in vivo* experiments, Dunnett's test was used. *P *< 0.05 was considered statistically significant. Error bars indicate standard deviations.

## Results and Discussion

### CDHP treatment reduces cell migration in basal‐like breast cancer

**Figure 1:**
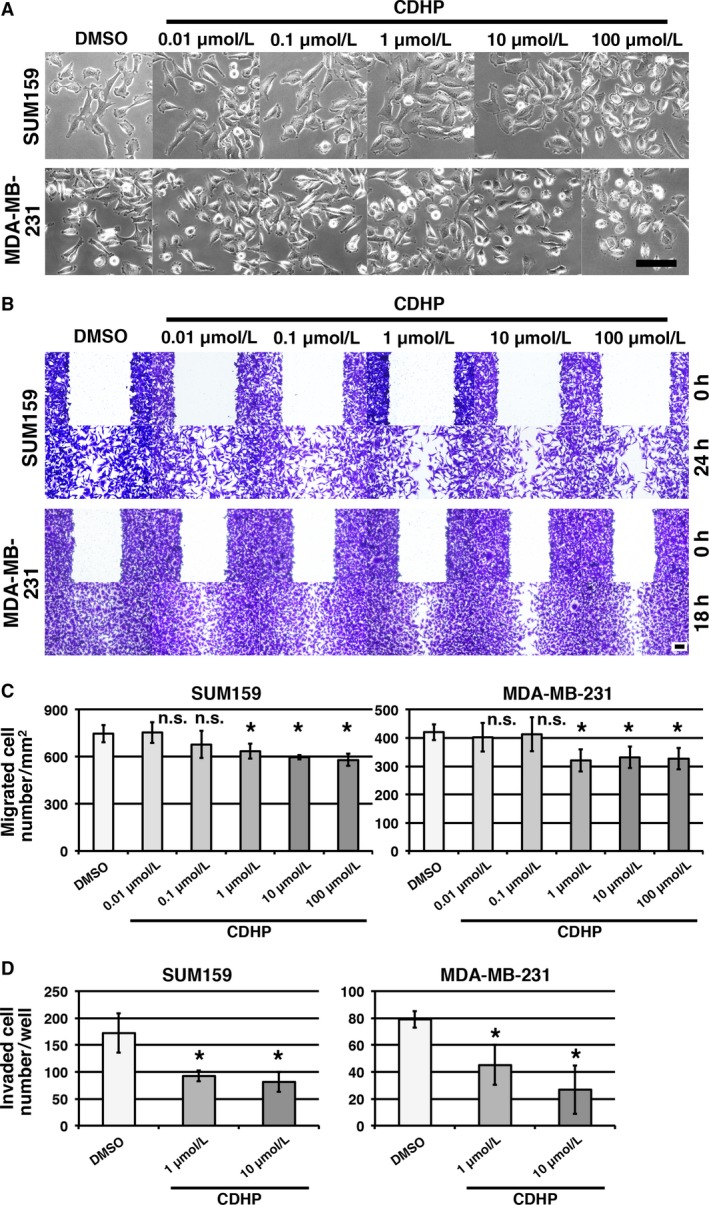
5‐Chloro‐2,4‐dihydroxypyridine (CDHP) treatment reduces cell migration (A) Images of CDHP‐treated cells are shown. Cells were cultured with CDHP for 3 days. (B) Wound healing assays were performed to analyze cell migration. Migrated cells are observed in scratched areas at 24 and 18 h in SUM159 and MDA‐MB‐231 cells, respectively. (C) The number of migrated cells were counted (*n* = 5 scratched areas). Inhibition of migration was observed in the cells treated with 1, 10, and 100 *μ*mol/L CDHP. (D) The number of invaded cells were counted (*n* = 4). Scale bars indicate 100 *μ*m. *P < 0.05 compared to DMSO controls (Dunnett's test).

Cancer cells migrate from the primary tumor to other organs during metastasis. In cell culture, morphological changes reflect the alteration of migratory ability. To determine whether CDHP alters cell migration, we treated the basal‐like breast cancer cell lines SUM159 and MDA‐MB‐231 with CDHP, and observed their morphology. In the DMSO control, basal‐like breast cancer cells showed an elongated morphology, and no remarkable change was observed in the cells treated with 0.01 and 0.1 *μ*mol/L CDHP (Fig. 1A). In the 1, 10, and 100 *μ*mol/L CDHP treatment groups, the cells were shrunken (Fig. 1A), suggesting that CDHP treatment alters cell migration.

To investigate the effect of CDHP treatment on cell migration, we performed wound healing assays. In the DMSO control, scratched regions were closed after 24 and 18 h in SUM159 and MDA‐MB‐231 cells, respectively (Fig. 1B). Cells treated with 1, 10, and 100 *μ*mol/L CDHP showed reduced migration, whereas the migration of 0.01 and 0.1 *μ*mol/L CDHP‐treated groups were not significantly changed compared to the DMSO control group (Fig. 1B and C). In addition, we performed the invasion assays. CDHP‐treated groups showed reduction in invasion ability (Fig. 1D). These results indicate that CDHP treatment reduces migration and invasion in basal‐like breast cancer cells.

**Figure 2:**
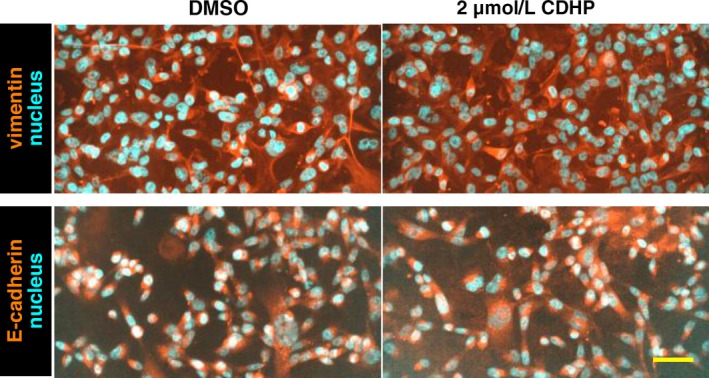
5‐Chloro‐2,4‐dihydroxypyridine treatment does not change the expression of mesenchymal and epithelial markers Immunostaining images of vimentin, a mesenchymal marker, and E‐cadherin, an epithelial marker, are shown. MDA‐MB‐231 LLM cells were used. Nucleus was counterstained with DAPI. Scale bar indicates 200 *μ*m.

For further study, we immunostained cells with antibodies for vimentin, a mesenchymal marker, and E‐cadherin, an epithelial marker, because alteration of mesenchymal and epithelial states changes cell morphology. However, we did not observe remarkable alteration of vimentin and E‐cadherin signals between DMSO control and CDHP‐treated groups (Fig. 2). This suggests that CDHP treatment does not change mesenchymal and epithelial states in basal‐like breast cancer cells.

### CDHP treatment reduces the number of nascent adhesions

In migratory cells, the formation of new focal adhesions moves a cell forward. Therefore we hypothesized that CDHP affects focal adhesion formation. During focal adhesion formation, paxillin localizes adhesion complexes from the stage of nascent adhesion formation to focal adhesion maturation, whereas vinculin localizes adhesion complexes at the stage of focal adhesion maturation 2. In an adhesion complex, paxillin is phosphorylated by focal adhesion kinase 14.

**Figure 3:**
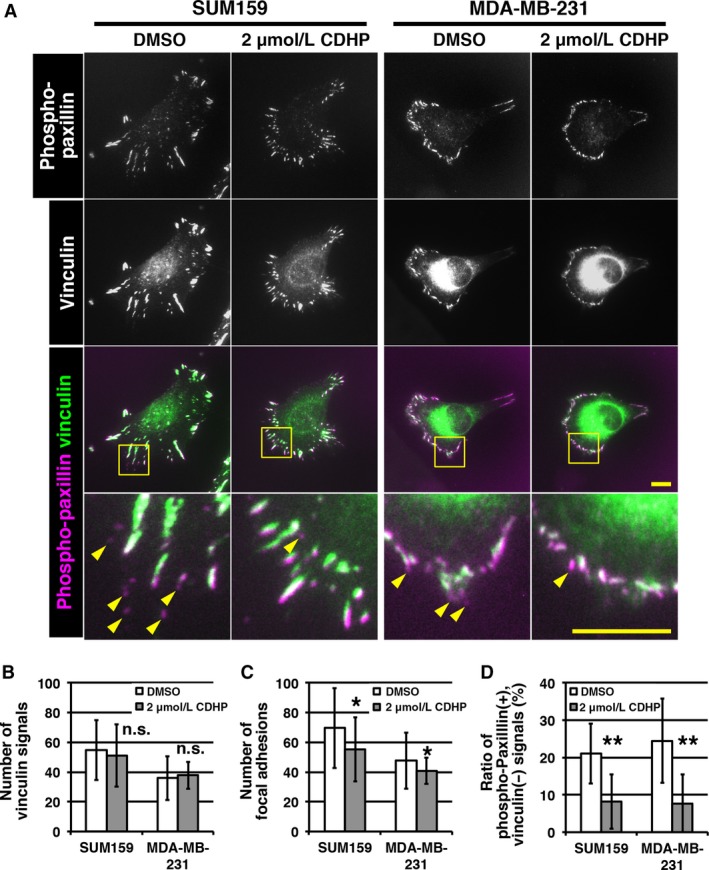
5‐Chloro‐2,4‐dihydroxypyridine (CDHP) treatment reduces the number of nascent adhesions (A) Images of double immunostaining for phospho‐paxillin and vinculin are shown. Cells were cultured with 2 *μ*mol/L CDHP for 3 days. Phospho‐paxillin/vinculin double‐positive focal adhesions have white signals in overlaid images. Yellow arrowheads indicate phospho‐paxillin positive, vinculin negative signals. (B–D) The numbers of vinculin‐positive adhesions (B) and total adhesions (C) were counted. The ratio of phospho‐paxillin single‐positive adhesions to total adhesions was calculated (D). SUM159 cells treated without or with CDHP (*n* = 32 and 41, respectively) and MDA‐MB‐231 cells treated without or with CDHP (*n* = 40 and 37, respectively) were analyzed. Scale bars indicate 10 *μ*m. * and ** indicate *P *< 0.05 and *P *< 0.01, respectively, compared to DMSO controls (Student's *t*‐test).

To analyze the effect of CDHP on focal adhesion formation, we performed immunostaining with antibodies for phosphorylated paxillin (phospho‐paxillin) and for vinculin. Focal adhesion signals are observed as dots at the edge region of a cell. A single‐positive immunostaining signal of phospho‐paxillin indicates nascent adhesion, and a double‐positive phospho‐paxillin/vinculin signal indicates mature focal adhesion. In our staining, the DMSO control cells showed phospho‐paxillin single‐positive and phospho‐paxillin/vinculin double‐positive adhesion signals (Fig. 3A). In the cells of CDHP‐treated groups, the number of phospho‐paxillin single‐positive signals was reduced, suggesting that CDHP affects focal adhesion formation (Fig. 3A).

When counting the number of signals per cell, the number of vinculin‐positive signals was not affected by CDHP treatment (Fig. 3B). The total adhesion number was determined by counting the number of phospho‐paxillin signals, and this number was reduced by CDHP treatment (Fig. 3C). The ratio of phospho‐paxillin single‐positive signals was significantly reduced (Fig. 3D). These data indicate that CDHP treatment reduces the number of nascent adhesions. Our results showed that CDHP treatment impairs focal adhesion formation, which may result in a reduction in cell migration.

### CDHP treatment inhibits lung metastasis

**Figure 4:**
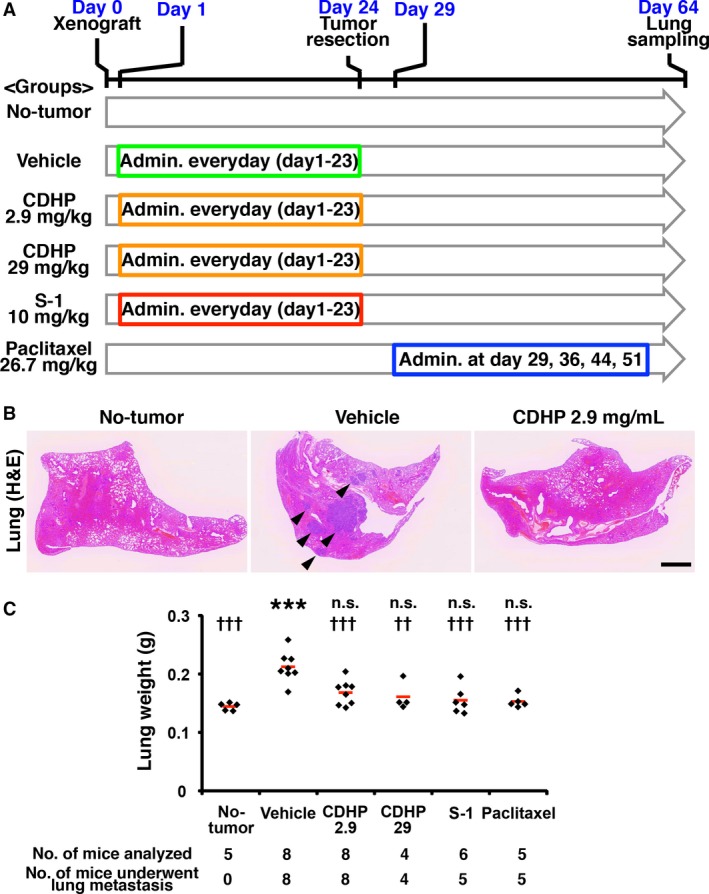
5‐Chloro‐2,4‐dihydroxypyridine inhibits lung metastasis (A) The design of the *in vivo* metastasis assay is depicted. (B) H&E staining of harvested lungs is shown. Metastatic foci were observed in the vehicle control (arrowheads). (C) Lungs weights are graphed. The weight of each lung is plotted, and the red bars indicate the mean value of each group. Scale bar indicates 1 mm. ***P < 0.001 compared to no‐tumor controls (Dunnett's test). ^††^ *P *< 0.01, ^†††^ *P *< 0.001 compared to vehicle control (Dunnett's test).

To analyze the effect of CDHP on metastasis *in vivo*, we used a highly lung‐metastatic subline of MDA‐MB‐231, named MDA‐MB‐231 LLM 13. Tumor tissue of the xenografted site was excised on day 24. On day 64, lungs were harvested, and whole lung weight was measured to evaluate metastasis. Lung weight on day 64 reflects the frequency of metastasis that occurred from day 0 to day 24 (Fig. 4A). No obvious metastasis in other organs was observed.

We used 0.5% hydroxypropyl methylcellulose as the vehicle control with the concentration of 10 mL/kg. CDHP was administered with a final dosage of 2.9 mg/kg, which corresponds to the amount of CDHP in the mice that were administered 10 mg/kg of S‐1. In addition, we used CDHP at 29 mg/kg, which is the concentration at which no weight loss was observed. Ten mg/kg S‐1 and 26.7 mg/kg paclitaxel were used as positive controls. Vehicle, CDHP or S‐1 was administered every day from day 1 to day 23. Paclitaxel was administered on days 29, 36, 44, and 51 to control the reduction in metastatic foci by drug administration. No body weight loss was observed in any group on day 24.

There was no significant difference in the weights of the tumors from the xenografted sites between the vehicle and CDHP‐treated groups (1.322 g ± 0.580, *n *= 8 in vehicle, 1.443 g ± 0.848, *n *= 8, *P *> 0.05 vs. vehicle in CDHP 2.9 mg/kg, and 1.304 g ± 0.738, *n *= 4, *P *> 0.05 vs. vehicle in CDHP 29 mg/kg). In the lung tissue, there were metastatic foci in the vehicle control, and reduced lung metastasis was observed in the CDHP‐administered group (Fig. 4B). However, in our observation, CDHP treatment did not completely prevent lung metastasis of MDA‐MB‐231 LLM cells, and lung metastasis was observed in all mice administered 2.9 and 29 mg/kg CDHP. Due to the generation of metastatic foci, the lung weight of the vehicle group was increased compared to the no‐tumor group (Fig. 4C). CDHP administration inhibited increase in lung weight compared to vehicle control, and the lung weights of the CDHP‐administered groups were not significantly increased compared to no‐tumor group (Fig. 4C), indicating that lung metastasis was suppressed by CDHP.

In this study, we discovered the new effect of CDHP for lung metastasis prevention in basal‐like breast cancer. In our *in vitro* experiments, the effect of CDHP was moderate. However, we observed remarkable inhibition of lung metastasis in CDHP‐administered mice. Given that *in vivo* experiments have more severe condition than *in vitro*, cells in *in vivo* seem to be more sensitive to the inhibitory effect of CDHP than *in vitro*.

CDHP is an inhibitor of DPD 15. A previous study has reported that DPD knockdown impairs the epithelial‐to‐mesenchymal transition in a normal breast epithelial cell line 16. However, in our observations, CDHP did not induce epithelial marker expression in basal‐like breast cancer cell lines (Fig. 2). This suggests that although DPD inhibition prevents mesenchymal transition of epithelial cells, it cannot induce epithelial transition in breast cancer cells that have mesenchymal features.

## Conclusions

S‐1 that contains CDHP is not only used in therapies for breast cancer, but also for other types of cancer. In clinical studies, S‐1 administration for adjuvant chemotherapy is effective in gastric and pancreatic cancers 9, 11, although a study for breast cancer is ongoing (UMIN000003969). These studies suggest that CDHP treatment is useful for lung metastasis prevention in various types of cancers. This study may contribute to the establishment of novel therapeutic approaches using CDHP, and to the development of future anticancer drugs for lung metastasis prevention.

## Conflict of Interest

JI is an employee of Kyoto University's Sponsored Research Program funded by Taiho Pharmaceutical Co., Ltd. MT received research funding from Taiho Pharmaceutical Co., Ltd. The funding source had no role in the study design, experiment, analysis, interpretation or writing the manuscript. HT, MN, and TT are employees of Taiho Pharmaceutical Co., Ltd.
